# Impact of percutaneous coronary intervention timing on 5-year outcome in patients with non-ST-segment elevation acute coronary syndromes. The ‘wait a day’ approach might be safer

**DOI:** 10.1007/s12471-016-0803-0

**Published:** 2016-02-09

**Authors:** Erik F.J. Oosterwerff, N. D. Fagel, T. Slagboom, J. G. P. Tijssen, J. P. Herrman, P. C. Smits, M. J. Suttorp, E. Ronner, G. J. Laarman, M. S. Patterson, G. Amoroso, M. A. Vink, R. J. van der Schaaf, F. W. A. Verheugt, R. K. Riezebos

**Affiliations:** Heart Centre, Department of Cardiology, Onze Lieve Vrouwe Gasthuis, 1090 HM PO Box 95500, Amsterdam, The Netherlands; Heart Centre, Department of Cardiology, Academic Medical Centre, Amsterdam, The Netherlands; Heart Centre, Department of Cardiology, Maasstad Hospital, Rotterdam, The Netherlands; Heart Centre, Department of Cardiology, St Antonius Hospital, Nieuwegein, The Netherlands; Heart Centre, Department of Cardiology, Reinier de Graaf Hospital, Delft, The Netherlands; Heart Centre, Department of Cardiology, TweeSteden Hospital, Tilburg, The Netherlands

**Keywords:** NSTE-ACS, Timing of PCI, Long-term outcomes

## Abstract

**Background:**

The OPTIMA trial was a randomised multicentre trial exploring the influence of the timing of percutaneous coronary intervention (PCI) on patient outcomes in an intermediate to high risk non-ST-elevation acute coronary syndrome (NSTE-ACS) population. In order to decide the best treatment strategy for patients presenting with NSTE-ACS, long-term outcomes are essential.

**Methods:**

Five-year follow-up data from 133 of the 142 patients could be retrieved (94 %). The primary endpoint was a composite of death and spontaneous myocardial infarction (MI). Spontaneous MI was defined as MI occurring more than 30 days after randomisation. Secondary endpoints were the individual outcomes of death, spontaneous MI or re-PCI.

**Results:**

No significant difference with respect to the primary endpoint was observed (17.8 vs. 10.1 %; HR 1.55, 95 % CI: 0.73–4.22, *p* = 0.21). There was no significant difference in mortality rate. However, spontaneous MI was significantly more common in the group receiving immediate PCI (11.0 vs. 1.4 %; HR 4.46, 95 % CI: 1.21–16.50, *p* = 0.02). We did not find a significant difference between the groups with respect to re-PCI rate.

**Conclusion:**

There was no difference in the composite of death and spontaneous MI. The trial suggests an increased long-term risk of spontaneous MI for patients treated with immediate PCI.

## Introduction

The optimal timing when using percutaneous intervention (PCI) in a non-ST-elevation acute coronary syndrome (NSTE-ACS) is still a matter of much debate. Early intervention may be beneficial in patients with NSTE-ACS as it could prevent a new myocardial infarction (MI). On the other hand, it could prove more harmful as it might lead to an increased incidence of periprocedural complications due to the thrombotic tendency of the culprit lesion. First-line medical pacification of the culprit coronary plaque may be beneficial but it requires time, during which a recurrent infarction may occur. The current guidelines recommend urgent (ESC guidelines definition: < 2 h) coronary angiography in patients with a very high risk, defined as: refractory angina, with associated heart failure, life-threatening ventricular arrhythmias, or haemodynamic instability. In addition, early (ESC guidelines definition: < 24 h) coronary angiography is recommended in patients with a GRACE risk score above 140 or with high-risk features, defined as a relevant rise or fall in troponin or dynamic ST or T-wave changes [1, 2].

The optimal timing of angiography and revascularisation in NSTE-ACS has been studied quite extensively. Yet the influence of the timing on PCI remains less clear due to the fact that previous trials chose to randomise the timing of the coronary angiography. The OPTIMA trial randomised patients with a suspected NSTEMI into immediate or delayed PCI after their eligibility had been established by acute coronary angiography [3].

In contrast to trials comparing routine versus selective invasive intervention, no long-term data from trials evaluating optimal timing of angiography have been published. Results of trials investigating routine versus selective invasive intervention in NSTE-ACS suggest that spontaneous MI in contrast to periprocedural MI increases long-term mortality [4–6]. Long-term outcomes should be known in order to decide the proper treatment strategy for patients presenting with NSTE-ACS.

In the current study, the 5-year clinical outcomes of death, MI and revascularisation of the OPTIMA trial are reported.

## Materials and methods

### Study design

The original study design and the results have been published previously [3] and the protocol was approved by the review board of all the participating centres. All patients gave informed consent.

The OPTIMA trial was a randomised multicentre trial exploring the influence of the timing of PCI on patient outcomes in an intermediate to high risk NSTE-ACS population. Follow-up was done on all the patients who had given their informed consent in the original OPTIMA trial. In cases where no information regarding the patient’s vital status or above-mentioned endpoints at 5-year follow-up could be retrieved from the patient file, the general practitioner and/or the local authorities were contacted. After it had been confirmed that the patient was still alive, the patient was contacted by telephone.

### Outcomes

The main outcome in the analysis was the combined endpoint of death and spontaneous MI. The secondary endpoints were the individual outcomes of death, spontaneous MI or re-PCI. Spontaneous MI was defined as: MI occurring more than 30 days after randomisation. The universal definition for MI was used [7]. The definition includes the detection of a rise and/or fall of troponin T with at least one value above the 99th percentile upper reference limit and with at least one of the following: symptoms of ischaemia, new or presumed new significant ST-segment–T wave (ST–T) changes or new left bundle branch block (LBBB), development of pathological Q waves on the ECG, imaging evidence of new loss of viable myocardium or new regional wall motion abnormality.

### Statistical analysis

The analysis was based on the intention to treat. Cumulative event rates were estimated using the Kaplan-Meier method. Hazard ratios (HRs) with 95 % confidence intervals (CIs) were obtained with Cox proportional hazards models and included the treatment strategy as the only covariate. Pre-specified subgroup analyses included gender, smoking, hypertension and diabetes. The GRACE RISK score was calculated for stratification purposes in all patients. Statistical analyses were applied using the Statistical Package for Social Sciences (SPSS version 15.0 for Windows, Chicago, Illinois).

## Results

**Fig. 1:**
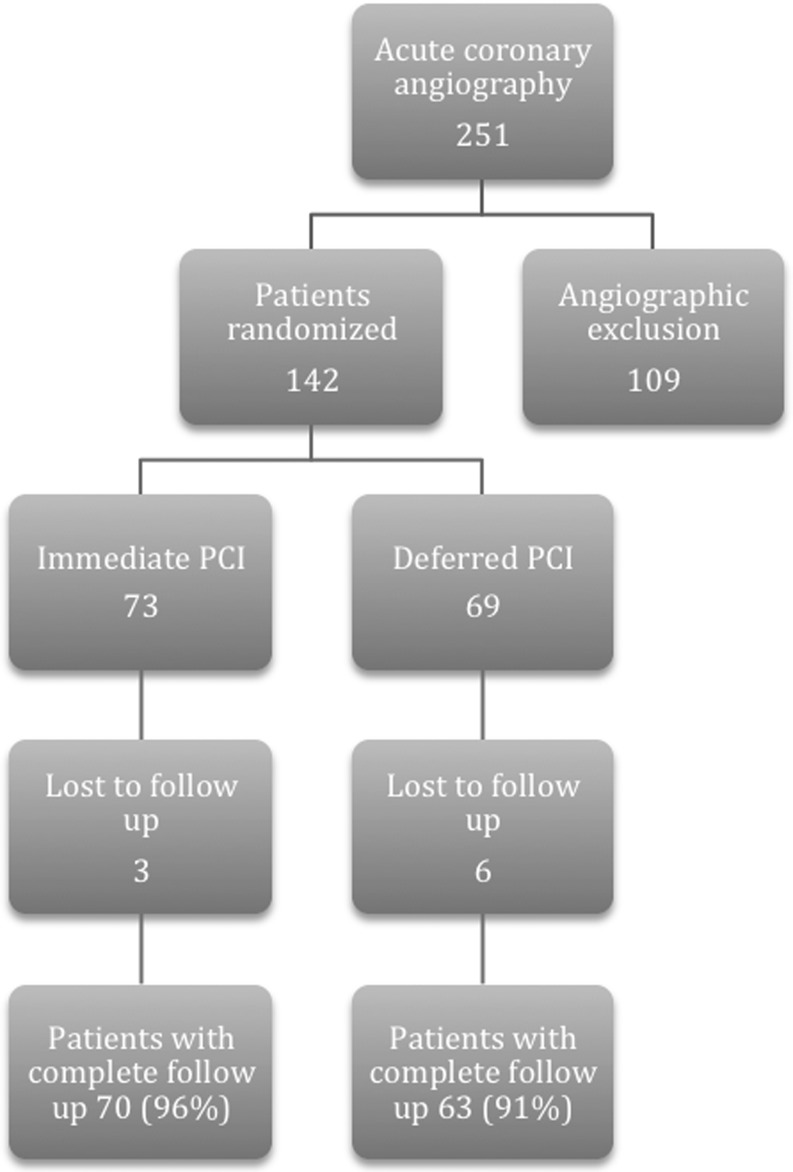
The reasons for (late) angiographic exclusion (*n* = 109) were: no significant CAD: *n* = 55, CABG: *n* = 27, instent restenosis: *n* = 9, clinically driven immediate PCI *n* = 8, culprit lesion not amenable to PCI: *n* = 6: chronic total occlusion: *n* = 4

From March 2005 until April 2007, 251 patients were enrolled in the OPTIMA trial. After undergoing immediate angiography, a culprit lesion amenable to PCI was identified in 142 patients. Patients were not randomised when angiography did not demonstrate significant coronary stenosis amenable for PCI, when coronary artery bypass grafting (CABG) was judged to be the preferred treatment, when the culprit lesion was instent restenosis or if the affected myocardial territory involved a chronic occlusion. These patients were treated according to institutional practices. The study randomised the patients amenable to PCI to an immediate (*n* = 73) or deferred (*n* = 69) coronary intervention. Table 1 shows the baseline characteristics, which were similar in the two groups, except for more previous CABG in the immediate group (11 vs. 1 %, *p* = 0.02) and the increased prevalence of hypertension in the immediate group (53 vs. 33 %, *p* = 0.03). The results of 30-day and 6-month follow-up were published previously [3] in which 74 (52 %) of the 142 patients showed multivessel disease. Of these, 26 % underwent treatment of two or more lesions leading to complete revascularisation in 74 % of the total number of patients. Treatment characteristics were equally distributed between the groups. Pharmacological treatment regimens were similar in both groups with over 95 % receiving aspirin and clopidogrel. Furthermore, the use of β-blocking agents and statins was high, indicating optimal medical treatment. For the complete lists of medication at admission we refer to the 30-day follow-up article [3]. The original study showed an increased rate of periprocedural MI in patients treated with immediate PCI as compared with patients with a PCI deferred for 24–48 h. Five-year follow-up data from 133 of the 142 patients could be retrieved (94 %). Three patients were lost to follow-up in the immediate group and six patients were lost to follow-up in the deferred group (Fig. 1).

**Table 1:** Baseline and patient characteristics

Characteristics	Immediate PCI (*n* = 73)	Deferred PCI (*n* = 69)	*p-* value
*Demographics*			
Male sex	51 (70)	51 (74)	0.6
Age (years), mean (SD)	63 (12)	62 (12)	0.8
Risk factors	41 (56)	34 (49)	0.4
Age > 60 years			
Known CAD	27 (37)	25 (36)	0.6
Diabetes mellitus	14 (19)	14 (20)	1.0
Hypertension	39 (53)	23 (33)	0.03
Smoking	28 (38)	27 (39)	0.8
Family history of IHD	32 (44)	29 (42)	0.8
Hyperlipidaemia	28 (38)	22 (32)	0.6
Peripheral artery disease	5 (7)	3 (4)	0.7
*Cardiac history*			
Previous MI	15 (21)	18 (26)	0.5
Previous PCI	20 (27)	13 (19)	0.2
Previous CABG	8 (11)	1 (1)	0.02
Previous CHF	1 (1)	1 (1)	0.3
*GRACE-risk score*			
≤ 140	59 (81)	54 (78)	0.7
> 140	14 (19)	15 (22)	0.7
*Coronary angiographic characteristics*			
Number of diseased vessels			
1	30 (41)	37 (54)	
2	33 (45)	22 (32)	0.3
3	10 (14)	9 (13)	
TIMI flow after PCI			
0–2	4 (5)	4 (6)	1.0
3	69 (95)	64 (93)	
Complete revascularisation	51 (70)	54 (78)	0.3
*PCI characteristics*			
No PCI performed	0 (0)	1 (1)	
Culprit lesions PCI	73 (100)	68 (99)	0.5
Multiple lesions treated	21 (29)	16 (23)	0.4

Data are expressed as number (%) unless stated otherwise.

*SD* standard deviation, *CAD* coronary artery disease, *MI* myocardial infarction, *PCI* percutaneous coronary intervention, *CABG* coronary artery bypass graft, *CHF* congestive heart failure, *IHD* ischaemic heart disease.

### Five-year follow-up

**Fig. 2:**
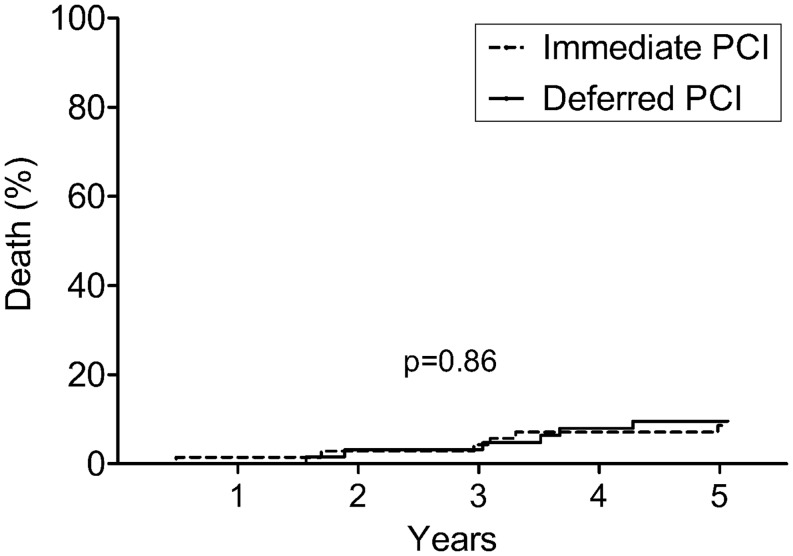
All-cause death (94 % follow-up rate)

No significant difference was found between the immediate and the deferred group with respect to the composite endpoint of death or spontaneous MI (17.8 vs 10.1 %; HR 1.55, 95 % CI: 0.73–4.22, *p* = 0.21). Mortality at 5 years was 8.22 % in the immediate group and 8.70 % in the deferred group (HR 0.90, 95 % CI: 0.29–2.80, *p* = 0.86, Fig. 2).

**Fig. 3:**
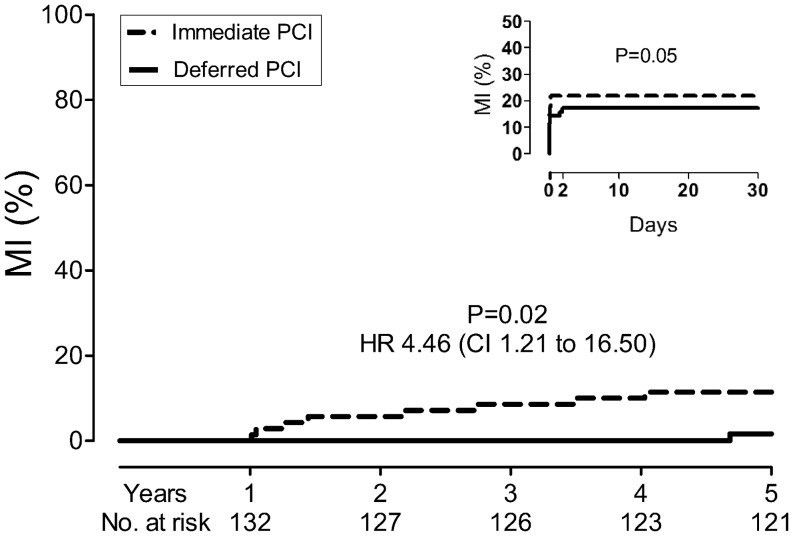
Event rate filtered for the index myocardial infarction (MI) during hospitalisation is shown in the main figure. Event rate of first 30 days of follow-up are presented on the right side. *HR* hazard ratio

However, a significant difference was found with respect to spontaneous MI at 5-year follow-up. In total, 8 patients in the immediate group and 1 patient in the deferred group experienced a spontaneous MI (11.0 and 1.4 %; HR 4.46, 95 % CI: 1.21–16.50, *p* = 0.02, Fig. 3). Since these events occurred at least 30 days after the index hospitalisation, this difference was irrespective of initial procedure-related myocardial infarction (Table 2). A sub-group analysis showed that most of the spontaneous MIs occurred in the index vessel (Table 3). There were no significant differences in the re-PCI rate at 5-year follow-up between the immediate and deferred group. The re-PCI rate was 6.8 % in the immediate group and 11.6 % in the deferred group (HR 0.53 95 % CI: 0.18–1.59, *p* = 0.26).

**Table 2:** Study endpoints at 5-year follow-up

	Immediate *n* = 73 (%)	Deferred *n* = 69 (%)	HR (CI)	*p-* value
Death or spontaneous MI	13 (17.8)	7 (10.1)	1.76 (0.73–4.22)	0.2
Death	6 (8.2)	6 (8.7)	0.90 (0.29–2.80)	0.9
Spontaneous MI	8 (11)	1 (1.4)	4.46 (1.21–16.5)	0.02
Re-PCI	5 (6.8)	8 (11.6)	0.53 (0.18–1.59)	0.3

Composite endpoint and individual endpoints.

*MI* myocardial infarction, *PCI* percutaneous coronary intervention, *HR* hazard ratio.

**Table 3:** Characteristics of spontaneous myocardial infarction

	Previous CABG	Index vessel Non-instent	Index vessel Instent restenosis	Spontaneous MI non index vessel related
Immediate *n* = 8	2	2	2	4
Deferred *n* = 1	–	–	1	–

Location of spontaneous myocardial infarction culprit lesion with respect to original region of interest during study index coronary catheterisation (and PCI).

*MI* myocardial infarction, *CABG* coronary artery bypass graft.

## Discussion

After 5 years, no differences appeared in the composite endpoint of death and spontaneous MI between the groups. However, the long-term follow-up of the OPTIMA trial suggests an increased risk of a spontaneous MI in those patients treated with an immediate PCI for their index NSTE-ACS. The pathophysiology behind this excess of spontaneous MI is most likely multifactorial.

NSTE-ACS represents 75 % of all ACS. It has been demonstrated that routine angiography and revascularisation after NSTE-ACS reduces mortality by 25 %, MI by 18 %, and re-hospitalisation for unstable angina by 31 % at mid-term follow-up [8].

In the past years, several studies have evaluated the influence of the timing of intervention in patients with NSTE-ACS. However, comparison of data and interpretation of the results remain difficult due to methodological differences between the studies [9–14].

The OPTIMA trial is the only trial that has actually randomised the timing of administering PCI instead of the timing of angiography. This approach is the only proper way to evaluate the influence of time on PCI outcome. However, it could be remarked that randomisation after an angiography could lead to bias in the selection of patients. As such, during the OPTIMA trial, great care was taken to include intermediate to high-risk patients and not to select on angiographic features. Arguably, a large number of patients with low TIMI flows at initial coronary angiogram were randomised [3]. The OPTIMA trial showed an increased rate of periprocedural MI in patients treated with immediate PCI. Most PCI-related infarcts were small, with little impact on myocardial function, and probably resulted from micro-emboli from the atherosclerotic plaque or disrupted thrombus particles, or formed during angioplasty or thrombotic side branch occlusions [15].

In order to decide the best treatment strategy for patients presenting with NSTE-ACS, long-term outcomes are essential. The long-term follow-up of the OPTIMA trial suggests an increased risk of spontaneous MI in those patients treated with an immediate PCI for their index NSTE-ACS. The long-term results show that half of the culprit lesions for spontaneous MI in the immediate group occurred within the index vessel. While the increase in re-infarction is -at least partly- explained by events originating from the index vessel, one could hypothesise a causal relationship between the timing of PCI during an acute event and the risk of a re-infarction later on.

For example, the increase in general coronary vascular tone that is often observed during an acute event could hamper the proper sizing of the stent. Incorrect sizing could lead to an increased risk of malpositioning and eventually cause a late ischaemic event [16]. Moreover, immediate PCI might have a higher risk of stenting non-significant lesions, due to overestimation of lesion severity in the very acute setting. Functional tests such as fractional flow reserve to assess the clinical significance of coronary artery stenosis of moderate/borderline severity do improve differentiation but were not used in the initial trial [17]. In addition, the high levels of coagulation and platelet activation that accompany an acute event are thought to increase the risk of periprocedural complications, including MI. Correspondingly, side branch occlusions and peripheral embolisation are more frequently seen in the hyper-acute setting [18–21]. While these complications increase, the long-term risks remain unknown. In accordance with a pooled analysis of three large ACS trials regarding the use of routine versus selective invasive procedures, the current study did not find a clear relation between the occurrence of periprocedural MI and the risk of late spontaneous MI or death [4]. Delaying the index procedure in order to allow for normalisation of the coronary vascular tone may result in a decrease in inflammatory, coagulation and platelet activity and might therefore be safer.

Some of the spontaneous MIs in the immediate group were not related to the index vessel. Deeper disease progression due to an imbalance in baseline characteristics such as a higher prevalence of previous CABG, hypertension and a mildly increased prevalence of dyslipidaemia in the immediate PCI group could correspond to the higher prevalence of late spontaneous MIs. There were no differences in the medical treatment post-PCI between groups; however, long-term medical compliance was not evaluated.

Whether a patient will develop an ST elevation or non-ST elevation ECG during ischaemia depends on the location of the culprit lesion and on the existence and extent of the collaterals. But sometimes STEMI and NSTE-ACS are syndromes without a sharp contrast: properties overlap. IJkema et al. stated that primary PCI might be considered regardless of the nature of the ST deviation [22]. Advancing developments in the pharmacological treatment of the ACS include the use of more potent statins, new anticoagulants and more reliable and potent platelet inhibitors [23]. In addition, novel intervention techniques including the use of fractional flow rate, intravascular ultrasound and optical coherence tomography guiding, could increase the safety and effectiveness of the index procedure. Moreover, a newer generation of drug-eluting stents and bioresorbable vascular scaffolds could prove safer in terms of long-term complications. The question remains whether these developments will provide a safer medium when treating those patients with immediate PCI. Future trials, such as the OPTIMA 2 (NTR3861), are currently recruiting patients in order to address this question.

For now, there appears to be no justifiable reason to rush patients to the cathlab when they can be stabilised medically. The ‘wait a day’ approach might even prove safer regarding both the risk of periprocedural and long-term spontaneous MI.

## Study limitations

The proposed number of patients included could not be achieved. In addition, there were 9 patients lost to follow-up. As such, the study was too underpowered to detect a difference in the clinical event rate at 5 years.

## Conclusion

The OPTIMA trial showed a higher incidence of periprocedural MI in NSTE-ACS patients treated with immediate PCI, as opposed to a ‘wait a day’ approach. Long-term follow-up showed no difference in mortality. However, the immediate strategy seems to be accompanied by an increased risk of late spontaneous MI as well. The exact mechanisms by which this excess is caused are currently unknown, but are most likely multifactorial in origin. Given the low numbers of total recurrent ischaemic events, these findings should be interpreted as hypothesis-generating. More long-term follow-up data of earlier and larger trials that randomised to timing of angiography in NSTE-ACS are eagerly sought after.
